# Multimethod Analysis of a Novel NiTi Rotary System: Cyclic Fatigue, Buckling Resistance, and Bending Tests

**DOI:** 10.1055/s-0045-1806949

**Published:** 2025-04-23

**Authors:** Alyne Rouse Rocha, Ana Grasiela Limoeiro, Iris Nogueira Seckler, Bárbara Rebeca Alves, Adriana Jesus Soares, Samuel Nogueira Lima, Victor Talarico Vieira, Marília Fagury Videira Marceliano-Alves, Wayne Martins Nascimento, Luis Cardoso Rasquin, Marcos Frozoni

**Affiliations:** 1 Departamento de Endodontia, Faculdade São Leopoldo Mandic, Instituto de Pesquisa São Leopoldo Mandic, Campinas, São Paulo, Brazil; 2 Department of Dentistry, Endodontics and Dental Materials, Bauru Dental School, University of São Paulo, Bauru, Brazil; 3 Department of Endodontics, Piracicaba School of Dentistry, State University of Campinas, Piracicaba, Brazil; 4 Department of Endodontics, Mário Juca University Center, Maceió, Alagoas, Brazil; 5 Department of Endodontics, Grande Rio University (UnigranRio), Rio de Janeiro, Brazil; 6 Department of Endodontics, Post- Graduate Program in Dentistry, Nova Iguaçu, Brazil; 7 Department of Endodontics, Maurício de Nassau University Centre (UNINASSAU), Rio de Janeiro, Brazil; 8 Department of Dental Research Cell, Dr. D. Y. Patil Dental College and Hospital, Dr. D. Y. Patil Vidyapeeth, Pune, India; 9 Department of Endodontics, Federal University of Bahia, Brazil

**Keywords:** endodontic, bending strength, fracture, resistance

## Abstract

**Objective:**

Instruments are susceptible to deformation and/or fracture, which may represent a failure in endodontic treatment. This study assessed the mechanical properties of nickel-titanium rotary instruments through cyclic fatigue, buckling resistance, and 45° bending tests.

**Material and Methods:**

One hundred and twenty rotary instruments were divided into three groups: Super Flexi Files Blue (SFB; 20/.04), TruNatomy Small (TN; 20/.04), and ProTaper Ultimate Shaper (PTU; 20/.04). They underwent cyclic fatigue tests at fixed and manufacturer-specified speeds, along with buckling and bending tests.

**Results:**

SFB files exhibited the greatest cyclic fatigue resistance with the longest time to fracture and highest number of cycles to failure (NCF), followed by TN and PTU. For PTU and TN, reducing rotation speed increased time to fracture and NCF, while SFB showed reduced values with speed increase. TN exhibited the highest buckling resistance, whereas speed significantly affected cyclic fatigue resistance, with SFB being the most resistant overall. TN demonstrated notable buckling resistance and flexibility.

**Conclusion:**

The rotational speed significantly affects the resistance of instruments to cyclic fatigue and that the SFB file is the most resistant file to cyclic fatigue.

## Introduction

The success of endodontic treatment depends on adequate cleaning and shaping of the root canal system, which can be safely performed by using mechanized nickel-titanium (NiTi) instruments. 1 These instruments significantly improve root canal shaping, reduce accidents, and increase the success rate of endodontic treatment. 2 Improvements in the metallurgy of NiTi alloys have enabled the development of a variety of new endodontic instruments leading to greater efficiency in canal shaping, better control of iatrogenesis, and consequently better clinical outcomes. Nevertheless, these instruments are still susceptible to deformation and/or fracture, which may represent a failure in endodontic treatment. 3 The main reasons for the occurrence of instrument fractures are cyclic fatigue fractures, torsion fractures, or a combination of both. 1 4

To overcome these problems, manufacturers have developed several strategies to improve the properties of the NiTi alloy. These include changes in kinematics, instrument design, and surface treatment that ensure better flexibility of the instruments and reduce grinding and transportation of curved root canals during preparation. 5

The Super Flexi Files Blue System (SFB; Rogin Dental, Shenzhen, Guangdong, China) is a system of rotating NiTi instruments with a trapezoidal cross-section, memory control, and heat treatment of the blue wire, which gives the instrument more flexibility and resistance. 6

The ProTaper Ultimate System (PTU; Dentsply Sirona, Charlotte, United States) has three different heat-treated alloys: M-wire (Slider), Gold-wire (SX, Shaper, F1, F2, F3), and Blue-wire (FX and FXL). It has a parallelogram cross-section with variable acute angles so that the cutting efficiency of each part of the file can be specifically adjusted depending on the expected workload in certain areas during endodontic preparation. 7

The TruNatomy system (TN; Dentsply Sirona, Charlotte, United States) has a square cross-section, a decentralized design, a variable taper, and has undergone a gold heat treatment which, according to the manufacturer, increases the elasticity and resistance to cyclic fatigue of the instruments by a factor of four, thus reducing the risk of fracture. 8 The TN instrument is made from a 0.8 mm thick NiTi wire instead of the 1.2 mm thick NiTi wire used in the manufacture of most instruments and then subjected to a special heat treatment. 9

The aim of this study was to evaluate the mechanical properties of the new endodontic instruments Super Flexi Files Blue 20/.04 compared to the previously studied instruments: ProTaper Ultimate Shaper and TruNatomy Small through two cyclic fatigue tests (at the same speed for the three systems and at different speeds according to the recommendations of each manufacturer), a 45° bending test and a buckling strength test of the endodontic instruments studied. The null hypotheses were as follows:

1. There would be no effect of rotational speed on cyclic fatigue resistance.
2. There would be no differences in resistance to cyclic fatigue between the instruments.
3. There would be no differences in bending strength between the instruments.
4. There would be no differences in buckling resistance between the instruments.

## Materials and Methods

**Fig. 1:**
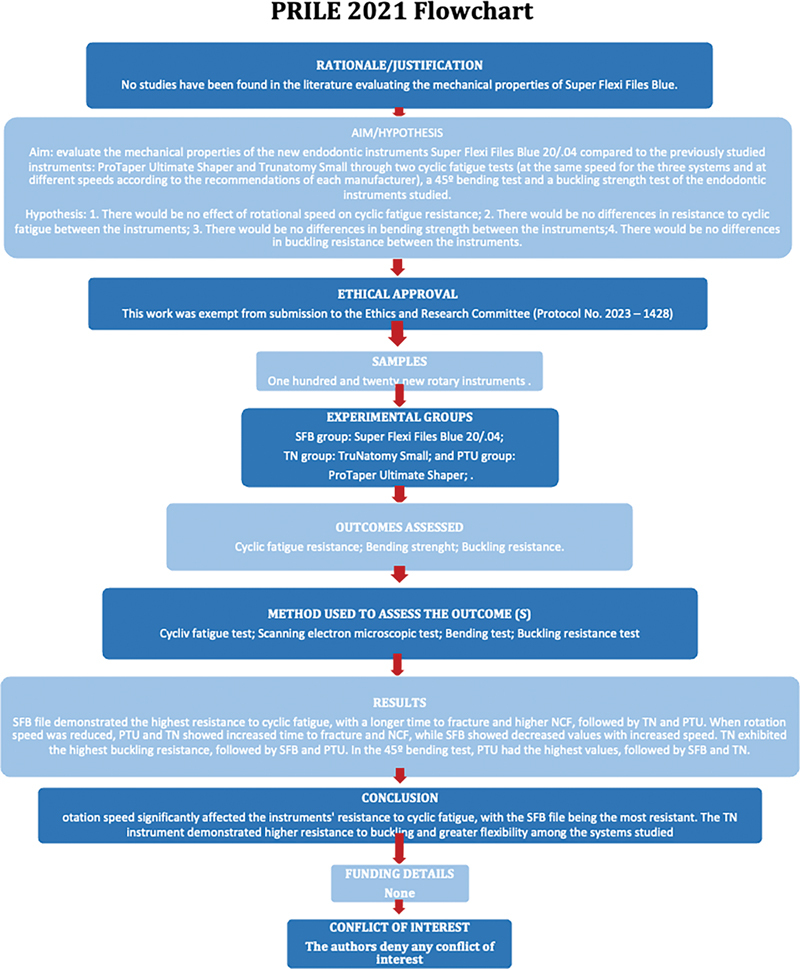
PRILE flowchart. PRILE, Preferred Reporting Items for Laboratory studies in Endodontology.

This study was exempt from submission to the Ethics and Research Committee (Protocol No. 2023–1428) and was conducted in accordance with Preferred Reporting Items for Laboratory studies in Endodontology (PRILE) 2021 guidelines 10 for laboratory studies ( Fig. 1 ). One hundred and twenty rotary NiTi instruments with a length of 25 mm, a tip size of #20, and a taper of 0.04 mm were used for this experiment. Each instrument was inspected under an operating microscope (Alliance Microscopia, São Carlos, Brazil) at 25× magnification for defects or deformations prior to the experiment and no instrument was discarded.

The instruments were randomly divided ( www.random.org ) into groups according to the tests performed ( *n* = 10): resistance to cyclic fatigue fracture at two speeds (fixed speed of 300 rpm and according to the speed recommended by the manufacturer), test buckling, and 45° bending test:

- SFB group: Super Flexi Files Blue (20/.04).
- TN group: TruNatomy Small (20/.04).
- PTU group: ProTaper Ultimate Shaper (20/.04).

### Sample Selection

**Fig. 2:**
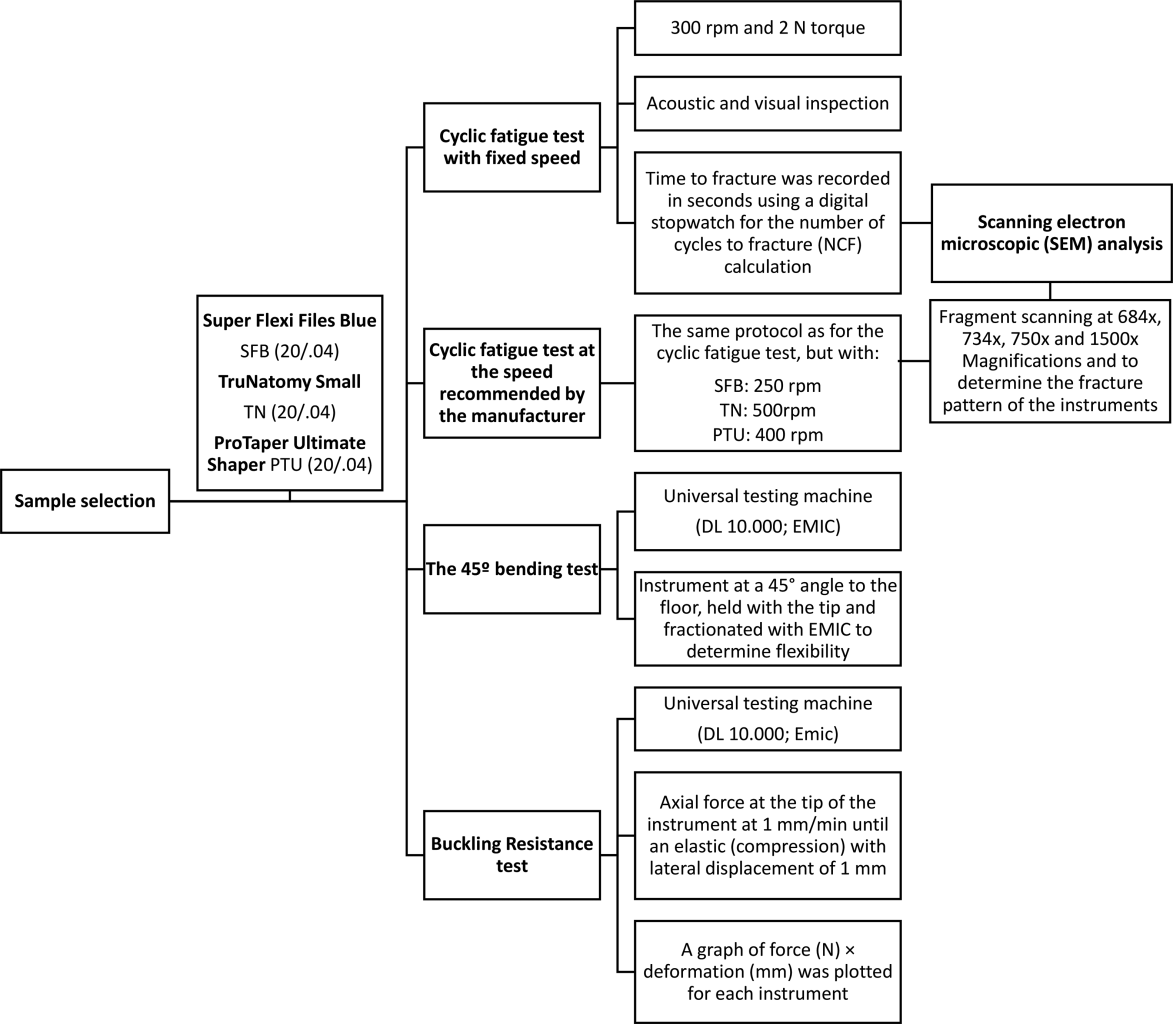
Methodology flowchart used in the study.

To calculate the most adequate sample size ( https://sample-size.net/ accessed on January 10, 2024), it was considered an alpha-level error of 0.05, a statistical power of 80%, and an estimated effect size and a significance level of 5%, the required sample sizes were calculated to be 7 and 9, respectively, to achieve statistical significance. To ensure sufficient statistical power, a final sample size of 10 files was chosen for each group. 11 All tests were performed by an experienced endodontist. Fig. 2 represents a flowchart that expresses the methodology used in the study.

### Cyclic Fatigue Test with Fixed Speed

**Fig. 3:**
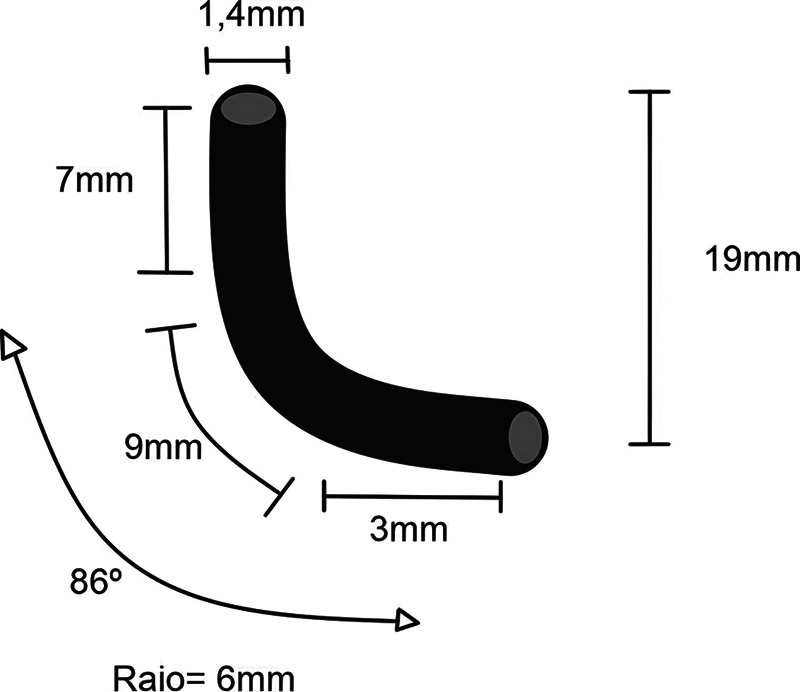
Schematic of the artificial canal used in this study.

The cyclic fatigue test ( *n* = 10) was performed on an apparatus consisting of an artificial metal canal made of a stainless-steel tube with a diameter of 1.4 mm and a total length of 19 mm. Between two straight segments with a length of 7 mm (cervical segment) and 3 mm (apical segment), a 9 mm long curved segment with a curvature of 86° and a radius of 6 mm—measured at the concave inner surface of the tube—was created ( Fig. 3 ).

Glycerine was used as a lubricant. The tested instruments were attached to a 6:1 reduction handpiece (Sirona Dental Systems GmbH, Bensheim, Germany) and were activated in static mode by a torque-controlled motor (VDW Silver; VDW GmbH) in a continuous clockwise rotation mode.

The fixed speed of this mechanical test was 300 rpm and a torque of 2 N for all tested systems. The test was performed at room temperature (30°C) according to the guidelines of the American Society for Testing and Materials 12 for the tensile test of super-elastic NiTi materials. Breakage was determined by acoustic and visual inspection. The time to fracture was recorded in seconds using a digital stopwatch, according to the method described in a previous study. 7 The number of cycles to fracture (NCF) was calculated for each instrument by multiplying the fracture time in seconds by the rotation speed (rpm) and dividing by 60 (seconds per minute), according to the following formula:

NCF = Time to fracture (sec) × Rotation speed (rpm)

60 seconds

### Cyclic Fatigue Test at the Speed Recommended by the Manufacturer

The second cyclic fatigue test ( *n* = 10) in the same way as the previous test but at speeds as recommended by the manufacturer: SFB: 250 rpm; TN: 500 rpm; PTU: 400 rpm.

### Scanning Electron Microscopic Analysis

**Fig. 4:**
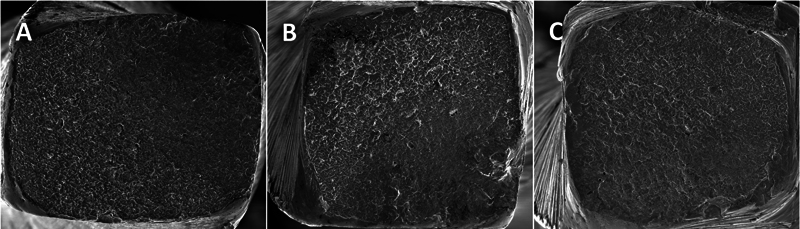
Representative images after analysis of the fractured surfaces of the instruments by SEM. ( **A** ) Super Flex Files Blue; ( **B** ) TruNatomy; ( **C** ) ProTaper Ultimate. Magnification: 684 × . Source: author. SEM, scanning electron microscopy.

After completion of the cyclic fatigue experiments, the fragments were cleaned in an ultrasonic bath to remove debris and dried in an oven at 37 °C for 24 hours. Three samples from each group were examined in a scanning electron microscope (SEM) to obtain photomicrographs of the fractured surfaces at different magnifications (684 × , 734 × , 750 × , and 1,500 × ) and to determine the fracture pattern of the instruments ( Fig. 4 ).

### The 45° Bending Test

The 45° bending test ( *n* = 10) was performed in accordance with the international standards ISO 3630-1 (1992). This mechanical test aimed to measure the 45° bending resistance of the endodontic instruments to evaluate their flexibility. The greater the bending resistance force, the less flexible the instrument and the greater its rigidity. The 45° bending test was performed with a universal testing machine (DL 10.000; EMIC, São José dos Pinhais, Brazil). Each instrument was attached to the motor head by the cable and was positioned at an angle of 45° to the ground, while its apical part, 3 mm from the tip, was attached to a 30 cm long wire.

The wire was connected to the universal testing machine and was loaded with a force of 20 N and pulled at a constant speed of 15 mm/min until the moment of maximum deflection (flexion) of the instrument.

This test provided a graph of the force (gf) versus the displacement of the instrument in millimeters, making it possible to measure the resistance to the elastic bending of the tip of the endodontic instrument. The greater the force used for this displacement, the less flexible the instrument is.

### Buckling Resistance Test

For the buckling resistance test ( *n* = 10), the instruments were coupled perpendicular to the floor to a universal testing machine (DL 10.000; Emic). The instruments were attached by cable to a rod attached to the head of the universal testing machine. The tip of the instrument was placed in contact with the bottom of a small cavity prepared in an aluminum plate. This cavity was machined with a spherical drill with a diameter of 1 mm and a depth of 0.5 mm. The load cell used had a value of 20 N and a force was applied to the tip in the axial direction at a rate of 1 mm/min until an elastic (compression) lateral displacement of 1 mm occurred. During the buckling test, a graph of force (N) × deformation (mm) could be plotted for each instrument. The maximum force required to cause an elastic lateral deformation (elastic displacement) of the instrument by 1 mm was recorded and considered as buckling resistance.

### Statistical Analysis

Two-criteria analysis of variance and Tukey tests were used to examine the time and number of cycles to instrument fracture and the effects of use at the manufacturer's recommended speed compared to the programmed speed (300 rpm). One-criterion analysis of variance and Games–Howell test were used to compare the instruments for flexural strength values. For buckling resistance data that followed a nonnormal distribution, the Kruskal–Wallis and Dunn tests were used. Statistical calculations were performed using the program SPSS 23 (SPSS Inc., Chicago, Illinois, United States) at a significance level of 5%.

## Results

### Cyclic Fatigue Test at the Speed Recommended by the Manufacturer

The time and NCF were significantly higher for the SFB 20/.04 instrument than for the TN Small, which in turn showed higher values than the PTU Shaper instrument ( Table 1 ).

**Table 1:** Means and standard deviations for time to fracture, number of cycles to fracture, flexural strength, and buckling resistance, according to the nickel-titanium file

Evaluated aspect		Super Flexi Files Blue	TruNatomy	ProTaper Ultimate
**Cyclic fatigue**	Time to fracture (sec)	319.7 (67.4) Aa (250 rpm)	64.6 (9.1) Bb (500 rpm)	53.6 (10.1) Cb (400 rpm)
		201.2 (29.7) Ab (300 rpm)	122.5 (21.7) Ba (300 rpm)	80.3 (7.3) Ca (300 rpm)
	Number of cycles to fracture	1332.2 (281.0) Aa (250 rpm)	538.4 (75.9) Bb (500 rpm)	357.4 (67.5) Cb (400 rpm)
		1006.0 (148.4) Ab (300 rpm)	612.5 (108.6) Ba (300 rpm)	401.5 (36.4) Ca (300 rpm)
**Strength**	Buckling (gf)	117.1 (6.1) B	177.0 (25.0) A	119.6 (12.4) B
	Bending (gf)	149.1 (16.7) B	94.1 (6.5) C	237.6 (12.8) A

Abbreviations: gf, gram-force; sec, seconds.

Note: Averages followed by different capital letters indicate a statistically significant difference between instruments (comparison within each line). For the data on times and number of cycles to fracture, distinct lowercase letters indicate a significant difference between the values measured at the speed indicated by the manufacturer and at 300 rpm (comparison within each column). For all aspects evaluated: *p* < 0.001.

### Cyclic Fatigue Test with Fixed Speed

The time and NCF were significantly higher for the SFB instrument than for the TN Small instrument, which in turn had higher values than the PTU Shaper instrument ( Table 1 ). It was also observed that the TN and PTU instruments achieved an increase in time and NCF when they reduced their speed from 500 rpm and 400 rpm to 300 rpm, respectively. For the SFB instruments, whose speed is specified by the manufacturer as 250 rpm, the use of 300 rpm led to a significant reduction in time and NCF ( Table 1 ).

### SEM Analysis

**Fig. 5:**
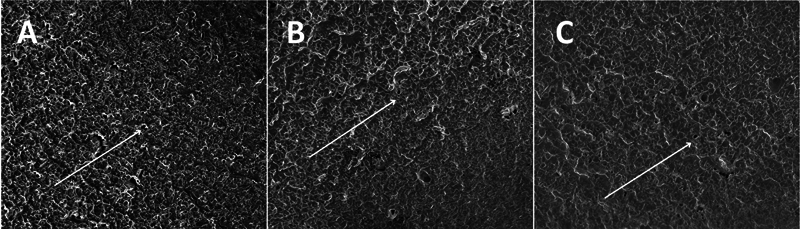
Representative images after analysis of the fractured surfaces of the instruments by SEM. The fractured surfaces show ductile-type morphological characteristics with numerous simple dimples (arrows). ( **A** ) Super Flexi Files Blue; ( **B** ) TruNatomy; ( **C** ) ProTaper Ultimate (1,500× magnification). SEM, scanning electron microscopy.

The samples of each group were examined in the SEM to obtain micrographs of the fractured surfaces at different magnifications and to determine the fracture pattern of the instruments ( Fig. 5 ).

### Test of the Buckling Resistance

The buckling resistance ( *p* < 0.001) was significantly higher for TN. There was no significant difference between the SFB and PTU instruments ( Table 1 ).

### The 45° Bending Test

Finally, for bending strength, the PTU instruments had significantly higher values ( *p* < 0.001) than the SFB instruments, which in turn had higher values than the TN instruments ( Table 1 ).

## Discussion

In this study, the mechanical properties of the SFB, TN, and PTU instruments were investigated. As part of these tests, the resistance to cyclic fatigue was analyzed in two situations: (1) at the speed recommended by the manufacturer and (2) at a fixed speed of 300 rpm, with the aim of isolating the speed factor and determining its influence on the resistance of the instruments to cyclic fatigue.

The cyclic fatigue tests were performed at room temperature (20°) and in the static model to reduce variables such as the amplitude of the axial movements of the instruments in the dynamic model and to obtain a precise position in the artificial root canal in the stainless-steel block according to the American Society for Testing and Materials. 12

In the two cyclic fatigue tests performed, the SFB instrument showed greater resistance to cyclic fatigue than the TN instrument, which was better than the PTU instrument, with a significant difference between them. The SFB instrument has a constant taper and a trapezoidal cross-section, while the PTU instrument has a progressive taper and a parallelogram cross-section, and the TN instrument has a regressive taper and a square cross-section. 13 14 The combination of the taper and geometry of the SFB, in addition to its heat treatment with blue wire, could explain the better performance of this instrument in the cyclic fatigue resistance tests performed, regardless of the speed used. 13

The point of maximum curvature of the metal device on which the test was performed is about 7 mm from the apical part. The SFB instrument has a diameter of approximately 0.48 mm in D7 (constant taper of 0.04) and thus has the smallest diameter in this segment among the instruments tested, which results in greater flexibility. Studies show that the smaller the cross-section, the greater the flexibility of the instrument and its resistance to cyclic fatigue. 15 16 17

Another difference in the SFB instruments is the heat treatment of the blue wire, which differs from the heat treatment of the gold wire in the TN and PTU instruments. The manufacturers do not provide any specific information, except that their instruments are heat-treated. Therefore, instruments with similar dimensions made from alloys with different heat treatments are expected to have different mechanical properties. Wires with blue heat treatment have a greater resistance to cyclic fatigue and a higher degree of twisting under torsional loading. 18 19 Although there is no detailed information on the manufacturing process of the SFB, heat treatments that color the surface of the instrument blue have been associated with a significant improvement in resistance to cyclic fatigue. 20 A study comparing ProFile instruments with and without heat treatment concluded that blued instruments have a higher resistance to cyclic fatigue and a lower resistance to buckling and bending. 21

The results of the cyclic fatigue resistance tests also showed that the TN instrument was stronger than the PTU. This result can also be explained by the combination of the square cross-section and regressive taper of the TN instrument, while the PTU has a progressive taper and a parallelogram cross-section. 15 16 17

When the rotational speed of TN and PTU was reduced from 500 and 400 rpm to 300 rpm, respectively, TN and PTU significantly improved their performance. The opposite was true when the rotation speed for the SFB instrument was increased from 250 to 300 rpm; here there was a significant decrease in time and NCF. Rotational speed appears to have a major influence on cyclic fatigue, as increasing the speed tends to shorten the time to fracture. 5 22 Therefore, the first and second null hypotheses were rejected.

One factor that may have contributed to this difference in results may have been the degree of curvature of the canals in the different studies, as the instruments may have been exposed to different clinical conditions. In studies performed at body temperature (37°C), the TN showed high resistance to cyclic fatigue compared to other instruments. 23 24 25

The bending strength of the PTU instrument was greater than that of the SFB instrument, which in turn was greater than that of the TN. Therefore, the third null hypothesis was also rejected.

This result means that the TN is more flexible than the other instruments. This result can be explained by the fact that the TN instrument has a quadrangular cross-section and a regressive taper, 14 which is smaller than the parallelogram cross-section of the PTU instrument 13 and the trapezoidal cross-section of the SFB file, as the bending resistance can be influenced by the cross-section, taper, and alloy of the instrument. 26 27 Previous studies 15 16 27 have shown that the larger the cross-section, the lower the flexibility of the instrument.

Progressive variable tapering, as present in the PTU instrument, can reduce its flexibility compared to a similar instrument with constant taper, such as the SFB. 28 On the other hand, the regressive variable taper that characterizes the TN instrument helps to increase the flexibility of the instrument compared to a similar instrument with constant or progressive taper. 28 These findings are consistent with the results of this study.

It is known that the heat treatment of the alloy also influences the flexural strength 15 27 29 ; therefore, the thermomechanical treatment of the instruments aims to produce a super-elastic NiTi alloy containing mainly a martensitic phase that is stable under clinical conditions. 15 In the present study, TN and PTU heat-treated with gold wire and SFB treated with blue wire showed good results in terms of flexural strength of the tested heat-treated instruments.

An interesting discovery was made in this study during the bending test. Although it was expected that highly flexible instruments would perform better in the cyclic fatigue resistance test, the TN was the most flexible instrument but showed lower resistance to cyclic fatigue than the SFB. This seemingly contradictory result can be explained by the differences in the small diameter of the NiTi wire used to make the TN (0.8 mm), while the other instruments studied have a 1.0 mm wire. 30 Similar results were found when comparing TN with Genius Proflex and Vortex Blue, which are also made with a 1.0 mm diameter wire.

The size of the cross-sectional area can also influence the mechanical properties, with a larger horizontal cross-sectional area leading to higher bending and torsional stiffness. 15 27 Compared to austenitic instruments, heat-treated NiTi instruments have been reported to have higher resistance to cyclic fatigue, higher strength 20 and higher flexibility, and show lower bending stress in bending tests. 7 15 27

Despite the lower bending strength, NT showed better buckling resistance; therefore, the fourth null hypothesis was also rejected. If the instrument has good buckling resistance, it means that it can advance apically safely and efficiently, especially in atretic canals. 26 The greater buckling resistance of the TN can be explained by its smaller diameter, as the instrument with a smaller diameter has a lower chance of buckling. 26

The buckling resistance increases proportionally to the modulus of elasticity of the instrument, i.e., the stiffer the instrument (NiTi alloy), the greater its buckling resistance. 19 The lower buckling resistance of the SFB (blue) and PTU (gold) instruments can be attributed to their heat treatments, which make them more flexible and consequently less stiff.

Unfortunately, the results of the SFB system could not be compared with the literature, as it is a system that has only recently been launched on the market and no scientific publication on its mechanical properties has yet been found.

In the present study, SFB files exhibited the highest cyclic fatigue resistance with the longest time to fracture and highest number of cycles to failure. The literature indicates that using more resistant instruments in root canal preparation offers advantages, such as greater efficiency and safety during endodontic procedures, as they present a lower risk of fractures, 31 allowing precise canal preparation without deviating from their natural anatomy. They also ensure faster and more comfortable treatment for the patient. 32 SFB instruments are cheaper than the systems compared in the study, which can lead to a more cost-effective treatment for dental offices. 33 34 These improvements significantly contribute to the long-term success of endodontic interventions.

The conditions under which the tests in this study were carried out, the ambient temperature and the use of static model, represent limitations of the research. This is because these conditions may differ significantly from the real clinical environment, where both the temperature and the dynamics of the movements are different. Therefore, it is essential to conduct meticulous and comprehensive laboratory studies in the future to fill this knowledge gap and promote more effective clinical practices.

## Conclusion

Within the limitations of the work, it can be concluded that rotational speed significantly affects the resistance of instruments to cyclic fatigue and that the SFB file is the most resistant file to cyclic fatigue. The TN file proved to be the most resistant and flexible of the systems examined.
